# Institutional trust is a distinct construct related to vaccine hesitancy and refusal

**DOI:** 10.1186/s12889-023-17345-5

**Published:** 2023-12-12

**Authors:** Sekoul Krastev, Oren Krajden, Zoua M. Vang, Fernanda Pérez-Gay Juárez, Elizaveta Solomonova, Maya J. Goldenberg, Daniel Weinstock, Maxwell J. Smith, Esme Dervis, Dan Pilat, Ian Gold

**Affiliations:** 1 Integrated Program in Neuroscience, McGill University, Montreal, QC Canada; 2 Faculty of Medicine, University of British Columbia, Vancouver, BC Canada; 3 Department of Sociology, McGill University, Montréal, Québec Canada; 4 Department of Philosophy, McGill University, Montreal, QC Canada; 5 Neurophilosophy Lab, Department of Philosophy, Division of Social and Transcultural Psychiatry, McGill University, Montreal, QC Canada; 6 Department of Philosophy, University of Guelph, Guelph, ON Canada; 7 Faculty of Law, McGill University, Montreal, QC Canada; 8 School of Health Studies, Faculty of Health Sciences, Western University, London, ON Canada; 9 Department of Psychology, McGill University, Montreal, QC Canada; 10 The Decision Lab, Montreal, QC Canada; 11 Department of Philosophy & Department of Psychiatry, McGill University, Montreal, Canada

**Keywords:** Vaccine hesitancy, Public health, Health communication, COVID-19, Conspiracy theories, Institutional trust, Public trust in science, Global health, Vaccine uptake

## Abstract

**Background:**

Vaccine hesitancy is driven by a heterogeneous and changing set of psychological, social and historical phenomena, requiring multidisciplinary approaches to its study and intervention. Past research has brought to light instances of both interpersonal and institutional trust playing an important role in vaccine uptake. However, no comprehensive study to date has specifically assessed the relative importance of these two categories of trust as they relate to vaccine behaviors and attitudes.

**Methods:**

In this paper, we examine the relationship between interpersonal and institutional trust and four measures related to COVID-19 vaccine hesitancy and one measure related to general vaccine hesitancy. We hypothesize that, across measures, individuals with vaccine hesitant attitudes and behaviors have lower trust—especially in institutions—than those who are not hesitant. We test this hypothesis in a sample of 1541 Canadians.

**Results:**

A deficit in both interpersonal and institutional trust was associated with higher levels of vaccine hesitant attitudes and behaviors. However, institutional trust was significantly lower than interpersonal trust in those with high hesitancy scores, suggesting that the two types of trust can be thought of as distinct constructs in the context of vaccine hesitancy.

**Conclusions:**

Based on our findings, we suggest that diminished institutional trust plays a crucial role in vaccine hesitancy. We propose that this may contribute to a tendency to instead place trust in interpersonally propagated belief systems, which may be more strongly misaligned with mainstream evidence and thus support vaccine hesitancy attitudes. We offer strategies rooted in these observations for creating public health messages designed to enhance vaccine uptake.

**Supplementary Information:**

The online version contains supplementary material available at 10.1186/s12889-023-17345-5.

## Introduction

### Vaccine hesitancy

Vaccines are one of the most powerful public health tools available to humanity. Since their invention in the late eighteenth century by Edward Jenner [1], they have saved countless lives, drastically reducing smallpox, polio, measles, mumps and rubella, among others [2]. More recently, Watson and colleagues estimate that 14.4 million COVID-19-related deaths were prevented globally by vaccines between December 2020 and December 2021 alone [3]. Not only have vaccines been extremely effective at saving lives, they have done so at what have historically been extremely low risks of side effects [4]. However, despite overwhelming consensus that vaccines provide a net benefit, a significant (and by many measures, increasing) portion of the global population is vaccine hesitant [5]—a term with a debated definition, most recently described as “a state of indecisiveness regarding a vaccination decision.” [6].

A retrospective study of 290 surveys spanning 149 countries and 284,381 individuals showed that a significant portion of the global population does not agree that vaccines are safe, important or effective [7]—beliefs that are strongly at odds with scientific consensus. Vaccine hesitancy has a wide range of negative consequences, from the more obvious health effects on unvaccinated individuals who become infected, to the more subtle, but likely larger, consequences for increased infection rates, especially within the social circles of unvaccinated individuals. For example, de Miguel-Aribas and colleagues [8] modeled the effect of COVID-19 vaccine hesitancy in the US and found that for each one percent decrease in vaccine hesitancy, the primary and secondary effects resulted in an aggregate of 45 deaths per million inhabitants averted.

### Vaccine hesitancy is a highly heterogeneous construct

Before tackling the determining factors of vaccine hesitancy, it is important to note several demographic factors that make the generalization of vaccine hesitancy research something that ought to be done with care.

Firstly, vaccine hesitancy has been a prevalent and well studied phenomenon for decades. However, prior to the COVID-19 pandemic, it was mostly studied in the context of childhood vaccinations, where parents have been the primary decision makers [9]. The COVID-19 pandemic created a renewed research interest in vaccine hesitancy in the context of adult vaccination. While this research has its own particularities related to the pandemic, COVID-19 vaccine hesitancy has been shown to overlap with parental vaccine hesitancy in a more traditional context. For example, Roberts and colleagues [10] carried out a study of over 1000 individuals and found a strong correlation between general anti-vaccination beliefs and COVID-19 vaccine hesitancy. At the same time, it is likely that the two constructs differ. To further complicate this, work by Merkley & Loewen [11] shows that different degrees of hesitancy apply to different COVID-19 vaccines, with more hesitancy associated with the AstraZeneca and Johnson & Johnson vaccines compared to Pfizer's and Moderna’s vaccines. Similarly, Brewer and colleagues [12] found that parents were more hesitant about certain vaccines (e.g. human papillomavirus [HPV] compared to measles, mumps and rubella [MMR]) and that reasons for hesitancy differ between vaccines (e.g., concerns about side effects for HPV versus efficacy for MMR).

Secondly, it is important to note that while vaccine hesitancy exists globally, different regions express it very differently. A 2018 global survey of over 140,000 people [7] showed that attitudes toward vaccines, which are closely related to vaccine hesitancy, vary significantly among different parts of the world; for example 95% of people in South Asia agree that vaccines are safe, while that figure sits at 72% in North America and 50% in Eastern Europe. Similarly, while awareness of vaccines is relatively high globally (90%), it varies from 98% in Australia and New Zealand to just 44% in Southern Africa. Dubé and colleagues [13] carried out a study that interviewed immunization managers from 13 different countries, which showed that not only the rates but also the factors involved in hesitancy differ significantly across geographies.

Thirdly, while factors affecting vaccine hesitancy are generally studied across different demographic groups, these groups can vary significantly in their expression of vaccine hesitancy. For example, Fajar and colleagues [14] carried out a meta-analysis that included 58 studies and found that factors associated with a higher likelihood of hesitancy include being a woman, being 50 years of age or younger, being single, being unemployed, living in a household with five or more individuals, having an educational attainment lower than an undergraduate degree and having a non-healthcare related job.

### What causes vaccine hesitancy?

Vaccine hesitancy is a highly heterogeneous construct that, as others have pointed out, is grounded in a complex ecosystem of historical, sociocultural and psychological factors [15, 16] that vary between countries, communities and vaccines. Indeed, past research has proposed that vaccine hesitancy is better understood in a context-specific manner along a continuum rather than as a single binary measure [17] and is affected by myriad factors such as parenting styles, the role of media, public health policies, health professionals, individual decision making and political ideologies, historical context and socio-cultural norms.

Past research has described the predictors of vaccine hesitancy under the umbrella of broader behaviors – for example using the Health Belief Model (HBM) [18] and the Theory of Planned Behavior (TPB) [19]. The HBM is a theoretical framework aiming to explain a person’s health-related behaviors in terms of a combination of a perceived threat and the apparent efficacy of a behavior aiming to reduce that threat. In the context of vaccination, the HBM suggests that vaccine uptake is dependent on factors such as a person’s beliefs about the severity of the disease, their susceptibility to it, as well as the vaccine’s efficacy and safety. The TPB instead focuses on an individual’s attitudes, social norms and perceived behavioral control to explain health-related behaviors. In contrast to the HBM, the TPB emphasizes the influence of a person’s social network and their feeling of control over the vaccination decision.

While the HBM and the TPB have been applied to a broad number of health-related behaviors, including vaccination, recent research has focused on psychological constructs that are specific to vaccine-related attitudes and decisions. Standard accounts specific to vaccine hesitancy situate its causes in a wide array of factors, including knowledge and information, past experiences, perceived importance of vaccination, risk perception and trust, subjective norms, religious and moral convictions, communication and media, public health and vaccine policies and health professional recommendations [9]. Expanding on this research, several models have been proposed to explain the causes of vaccine hesitancy. For example, 1) the 3Cs model, developed by the World Health Organization's (WHO) SAGE group in 2014, whereby vaccine hesitancy is determined by complacency, convenience and confidence; 2) the 5As model, which includes access, affordability, awareness, acceptance and activation [20]; and 3) the 5Cs model, which expands on the 3Cs to include confidence, complacency, constraints, calculation and collective responsibility [21].

### Beyond informational models of vaccine hesitancy

Given the gap between scientific consensus on vaccine safety and effectiveness and hesitancy rates, there has been a deliberate and expensive effort by governments around the world to close this gap using clearer communication strategies. Lack of high quality information has been deemed such an issue that it has led the WHO to state we are fighting an “infodemic” [22]—i.e., that messaging strategies aiming to overcome lack of correct information about vaccines might be the front-line of our vaccination uptake efforts. Unfortunately, there is a growing amount of evidence that suggests communication strategies focused on filling an information gap have largely failed [9], leading some to suggest that approaching vaccine hesitancy from the assumption it is a gap in information or rationality takes the onus away from governments [16, 23].

Indeed, while misinformation is clearly related to vaccine hesitancy, evidence suggests the link may be complicated. As Goldenberg [16] has argued at length, the postwar reduction in vaccine acceptance in the industrialized north is more likely to be the result of an erosion of trust in institutions [16], and in medicine in in particular [24], due to factors such as a legacy of social exclusion [25], under-representation and unethical treatment of marginalized groups in health research [15] and historical trauma [16], which has led to a situation where scientific consensus is, for the vaccine hesitant, largely irrelevant. This situation is reminiscent of conspiracy thinking—another case where mainstream evidence is disregarded for a variety of reasons. In fact, a strong link between conspiratorial thinking and vaccine hesitancy has been documented, suggesting a possible overlap in the causal factors behind these phenomena. For example, Hornsey and colleagues [5] sampled over 5000 participants across 24 countries, showing a strong association between vaccine hesitancy and conspiratorial thinking. In fact, Stoler and colleagues [26] showed that belief in conspiracy theories was the biggest predictor of vaccine hesitancy.

As argued by Grasswick [27], the relationship between scientific communities producing that consensus and lay communities is such that epistemic merit is earned by more than simply “following the standards of normal science”. As is the case in individuals holding conspiracy beliefs, even in situations where good science practices are followed, if the producer or messenger of a particular insight is deemed untrustworthy, the message is likely to be ignored. This means that trust is a necessary (and perhaps sufficient) component of vaccine acceptance and a key factor in vaccine hesitancy, which has received insufficient attention in the past and has had limited impact on the shaping of public health strategies [28].

### Trust and vaccine hesitancy

A large body of literature exists on trust, from a wide variety of fields, yielding many possible definitions. One common definition is that trust is a “willingness to accept vulnerability based on positive expectations of the intentions or behavior of another” [29]. Trust is a complex relational practice happening within particular socio-political contexts [30]. In the context of group collaboration, trust has been shown to facilitate positive outcomes including information sharing and task performance [31]. Findings about trust and cooperation carry over to a vaccine context, where it is widely acknowledged that lack of trust is a key predictor of vaccine hesitancy [32], and is associated with lower vaccine uptake [33]. However, given the large number of actors—both other people (i.e., requiring interpersonal trust) and institutions (i.e., requiring institutional trust)—that a person may consider when making a vaccination decision, we know surprisingly little about how trust toward these various “others” relates to vaccine hesitancy.

### Interpersonal and institutional trust

There is a growing literature on the factors that affect interpersonal trust [34, 35]. An individual's behaviors can be understood through the ability to infer mental states of others, known as the Theory of Mind, using cues such as facial features, body language and eye contact [36]. In addition, an individual has a history of actions that can be attributed directly to them which can be translated into markers of trust such as honesty, reliability and competence [37]. Therefore, psychosocial signals offer a good explanatory model for trust toward individuals and small groups where interpersonal connections occur. However, they are less effective at explaining trust in institutions, where other factors related to connectedness and past experiences become more relevant [38, 39].

Institutions have been conceived of in various ways—through a structural–functional lens (i.e., as an interconnected system), as described by scholars including Peter Scott [40], and as a set of rules and goals put together by actors into a cohesive whole that has an identity by those such as John Meyer [41]. Whichever conception is used, institutions differ drastically from individuals in the ways they engender trust. For this reason, while past studies may be construed to refer to “trust” in a vaccine hesitancy context under the premise that it is a single construct, they are in fact referring to very different constructs that we would expect to behave differently. Indeed, interpersonal and institutional trust are distinct. While both result in the same behavior (i.e., a “willingness to accept vulnerability based on positive expectations of the intentions or behavior of another”), past studies have shown that institutional trust is more closely related to the concept of social identity and belonging [42], while interpersonal trust is more closely based on social appeal [43].

The decline in institutional trust during the latter half of the twentieth century and early twenty-first century, caused by a variety of factors such as political polarization, economic inequality, government inefficiency and social media echo chambers, has had a profound effect on vaccine hesitancy [16]. The effect of this decline has been heterogeneous across different communities, with minority communities who may have been the victims of mishandling of public health crises, historical mistreatment and marginalization, exhibiting higher levels of mistrust in institutions. Research shows that this erosion in institutional trust has affected vaccination decisions [44–47] in a way that is perhaps exacerbated in those communities. Indeed, a comprehensive review by Sapienza and Falcone [33] on the role of trust in COVID-19 vaccine hesitancy suggests a complex relationship between the two. For example, they found positive correlations between levels of trust in the COVID-19 vaccine and being male, being older, and having a higher level of income. In the context of institutional trust, they found that trust in government generally relates positively to COVID-19 vaccine acceptance—the one exception being trust in the Trump government, which had a negative relationship with COVID-19 vaccine acceptance. This example underscores and important distinction between studies that might be measuring trust in a specific institution (e.g. the current government at that moment) versus trust in institutions more generally.

A growing amount of research on vaccine hesitancy has focused on trust. While many past studies treat trust as a single construct or focus on measures of trust that relate to specific entities or institutions, “trust in institutions”, in a more general sense is likely to be a distinct construct that is particularly relevant to citizen behaviors, such as vaccination, that require engagement with large public agencies.

### Objectives and hypotheses

In order to gain a better understanding of institutional trust as a potential factor related to vaccine hesitancy, our present objective is to investigate the trust attitudes of vaccine hesitant and non-hesitant individuals in relation to interpersonal and institutional contexts. We hypothesize that vaccine hesitant individuals have distinct trust attitudes toward individuals and institutions. More specifically, we hypothesize that those with high levels of hesitancy-related attitudes and behaviors, as measured by several related constructs (COVID-19 vaccine hesitancy, COVID-19 vaccine concerns, general vaccine hesitancy, COVID-19 conspiracy thinking and COVID-19 vaccination status), are more likely to exhibit low levels of trust that are specific to an institutional context. In other words, we hypothesize that trust deficits reported in vaccine hesitant individuals by previous research are in fact deficits specific to institutional trust.

## Methods

### Sample and data gathering procedure

In collaboration with Environics Research [48], a Canadian polling and research firm, a representative group of 1541 Canadians aged 18 and older were randomly recruited by email invitation through either Dynata [49], the world’s largest first-party research data platform, or Asking Canadians) [50], Canada’s premier proprietary research panel.

Participants had already consented to participate and were asked to read and sign additional consent forms from McGill University and Environics Research that provided the information pertinent to the present study. Subsequently, participants were asked demographic questions, including age, gender, ethnicity, Indigenous status, province, education, and income. A summary table of sample characteristics can be found in Additional file 1: Appendix A.

During April and May of 2021, participants were sent a link to the survey after being contacted by Dynata and/or Asking Canadians. The entire survey was conducted online, and could be completed on a computer or mobile device (mobile phone or tablet). There was no time limit, and participants were informed they could withdraw from the study at any time or decline to answer any questions in the survey. Participants were not compensated given their pre-existing agreement with Dynata and/or Asking Canadians.

### Measures

Questions assessed levels of trust and four factors that have been previously found to relate to vaccine hesitancy: conspiratorial thinking, COVID-19 vaccine hesitancy, general vaccine hesitancy, and COVID-19 vaccination status. The instruments used can be found in Additional file 1: Appendix B.

#### Trust

Trust was measured using interpersonal and institutional trust self-report, based on OECD guidelines [51], which were previously used in the COVIDiSTRESS survey (*n* = 173 429 respondents in 48 countries) [52, 53] and its follow-up study (*n* = 15, 700) [54]. Measures of trust were split into two groups: *interpersonal trust*, composed of family, friends, acquaintances, classmates, co-workers and roommates; *trust in institutions*, composed of federal government, local government, WHO, healthcare system, police, scientists, physicians, mainstream media and pharmaceutical companies. Cronbach's Alpha for each of these groups, as determined using the Pingouin package on python, yielded an alpha of 0.802 for "interpersonal" and 0.894 for "institutional", which indicates a good level of internal consistency between the variables making up each of these composite measures.

#### COVID-19 vaccine hesitancy

In line with past work [55, 56], COVID-19 vaccine hesitancy was determined based on a combination of vaccination status and two other factors: for those who were vaccinated, we used the level of reported hesitancy prior to vaccination; for those who had not been vaccinated, we evaluated their vaccination intentions. This instrument (Section B1 of Additional file 1: Appendix B) allowed us to divide participants into groups based on the level of COVID-19 hesitancy that they exhibited. In particular, the following groupings were made; note that Group 1 includes two subgroups:

- 1a: Vaccinated & Non-hesitant
  - ◦ *Answered “Yes” to “Knowing that vaccinations against COVID-19 have begun, have you received the COVID-19 vaccination?”*
  - ◦ *Answered 1–3 (Not Hesitant to Neither hesitant nor Non hesitant) to “Thinking back, how hesitant were you about a COVID-19 vaccination prior to receiving one?*
- 1b: Unvaccinated & Non-hesitant
  - ◦ *Answered “No” to “Knowing that vaccinations against COVID-19 have begun, have you received the COVID-19 vaccination?”*
  - ◦ *Answered “Yes, I would get a vaccination as soon as one became available to me” to “When the COVID-19 vaccination becomes available to you, would you get vaccinated or not?”*
- 2: Vaccinated & Hesitant
  - ◦ *Answered “Yes” to “Knowing that vaccinations against COVID-19 have begun, have you received the COVID-19 vaccination?”*
  - ◦ *Answered 4–5 (Hesitant or Extremely Hesitant) to “Thinking back, how hesitant were you about a COVID-19 vaccination prior to receiving one?”*
- 3: Unvaccinated & Soft-Hesitant
  - ◦ *Answered “No” to “Knowing that vaccinations against COVID-19 have begun, have you received the COVID-19 vaccination?”*
  - ◦ *Answered “Not sure” or “Yes, I would eventually get a vaccination, but would wait a while first” to “When the COVID-19 vaccination becomes available to you, would you get vaccinated or not?”*
- 4: Unvaccinated & Hard-Hesitant
  - ◦ *Answered “No” to “Knowing that vaccinations against COVID-19 have begun, have you received the COVID-19 vaccination?”*
  - ◦ *Answered “No, I would not get a COVID-19 vaccination” to “When the COVID-19 vaccination becomes available to you, would you get vaccinated or not?”* 

For the purpose of our analysis, the four groups that resulted allowed us to distinguish between those with no COVID-19 vaccine hesitancy (defined as vaccinated or unvaccinated and non-hesitant) and those with COVID-19 vaccine hesitancy (vaccinated and hesitant, unvaccinated and soft-hesitant, or unvaccinated and hard-hesitant). The subgroup sizes for the four groups were as follows: 972 individuals were included in Group 1; 193 were included in Group 2; 168 were included in Group 3; 208 were included in Group 4.

#### COVID-19 vaccine concerns, general vaccine hesitancy & COVID-19 conspiratorial thinking

COVID-19 vaccine concerns, general vaccine hesitancy and conspiratorial thinking were measured using the instruments described in Additional file 1: Appendix B. For the purpose of t-tests, each outcome variable was split into two groups: a “hesitant” group, composed of everyone who scored above the median on that measure; a “non-hesitant” group, composed of everyone who scored at or below the median for that measure.The cutoff for the groups was as follows: 2.40 for conspiratorial thinking, 0.17 for COVID-19 vaccine concerns and 0 for general vaccine hesitancy. The sizes of the corresponding subgroups were as follows: 720 for the non-hesitant conspiratorial thinking group; 821 for the hesitant conspiratorial thinking group; 839 for the non-hesitant COVID-19 concerns group; 702 for the hesitant COVID-19 concerns group; 1422 for the non-hesitant general vaccine hesitancy group; 119 for the hesitant general vaccine hesitancy group. In addition, 1131 individuals were unvaccinated for COVID-19 and 410 were vaccinated.

### Data analysis

To investigate the relationship between interpersonal and institutional trust and the four outcome measures (conspiracy thinking, COVID-19 vaccine hesitancy, general vaccine hesitancy, and COVID-19 vaccination status), we calculated eta correlation ratios between our dependent and independent variables using the statsmodels package [57]. Eta correlation ratios were used instead of Pearson’s correlation ratios because of the ordinal nature of our variables. We then used a bootstrap method in order to assess the statistical significance of the differences between these correlations. Utilizing the bootstrap method, a non-parametric approach that imposes no assumptions about the underlying population distribution, enabled us to empirically estimate the sampling distribution of our statistics of interest [58]. This approach is particularly advantageous as it is robust against violations of normality assumptions, which can be a concern in traditional parametric tests. By employing the bootstrap method, we not only addressed potential concerns about the validity of our results but also provided a transparent and interpretable representation of the uncertainty surrounding our estimates. We also split each of the outcome variables into high and low groups based on their median score, and conducted two t-tests for each variable using the statsmodel package: one between interpersonal and institutional trust in the high group, and another in the low group. To enhance the robustness and validity of our statistical inferences, we applied the bootstrap method with scikit-learn, pandas, and numpy Python packages to resample the data with replacement, thereby obtaining distributions for the correlation coefficients and t-statistics.

## Results

Our results report on a sample of 1541 participants; sample characteristics are included in Additional file 1: Appendix A.

### Eta correlation ratio between trust in each entity and vaccine hesitancy measures

As a first step in our analysis, we created an eta correlation ratio matrix showing the relationship between trust in each of the entities and the five measures related to vaccine hesitancy (Table 1). The results showed that stronger eta correlations ratios were present for institutional entities across all five measures. In particular, the strongest correlation ratios were between trust in scientists and vaccination status (η = 0.564), trust in scientists & COVID-19 vaccine hesitancy (η = 0.498) and trust in the WHO and COVID-19 vaccination status (η = 0.457). A post-hoc examination of the net change in means across the trust categories was conducted for each variable pair, with relationships classified as either 'Increasing' or 'Decreasing', based on whether the mean of the predictor variable was higher for the last category of the trust variable than for the first. This allowed us to ascertain the direction of the effect, which was Negative for all 75 pairs.

**Table 1:** Matrix showing the relationship between trust in each specific entity and the five measures related to vaccine hesitancy, as quantified by the Eta Correlation Ratio. A post-hoc examination of the net change in means across the trust categories was conducted for each variable pair, with relationships classified as either 'Increasing' or 'Decreasing', based on whether the mean of the predictor variable was higher for the last category of the trust variable than for the first. This allowed us to ascertain the direction of the effect which was Negative for all 75 pairs

	**COVID-19 Vaccine Hesitancy**	**COVID-19 Vaccine Concerns**	**General Vaccine Hesitancy**	**COVID-19 Conspiracy Thinking**	**COVID-19 Vaccination Status**
Interpersonal Trust					
Trust family	.145	.134	.109	.133	.131
Trust Roommates	.140	.135	.086	.115	.160
Trust Friends	.146	.140	.109	.130	.193
Trust Classmates	.164	.155	.048	.122	.113
Trust co-workers	.161	.145	.066	.122	.142
Trust Acquaintances	.179	.159	.075	.158	.133
Institutional Trust					
Trust police	.208	.214	.109	.238	.178
Trust local govt	.308	.258	.197	.310	.329
Trust Pharma	.322	.264	.232	.333	.235
Trust Fed govt	.336	.277	.220	.342	.353
Trust Media	.319	.271	.230	.332	.356
Trust doctors	.347	.297	.263	.332	.363
Trust Healthcare system	.374	.292	.271	.367	.378
Trust WHO	.374	.326	.260	.377	.457
Trust scientists	.498	.414	.306	.432	.564

We next sought to investigate the association between high and low levels of COVID-19 vaccine hesitancy and varying degrees of interpersonal and institutional trust. Participants were categorized as hesitant and non-hesitant based on the method described above. Our analysis revealed that hesitant individuals displayed overall reduced trust (M = 3.06, SD = 0.70) compared to their non-hesitant counterparts (M = 3.54, SD = 0.58; t(1539) = -14.54, *p* < 0.001).

**Fig. 1:**
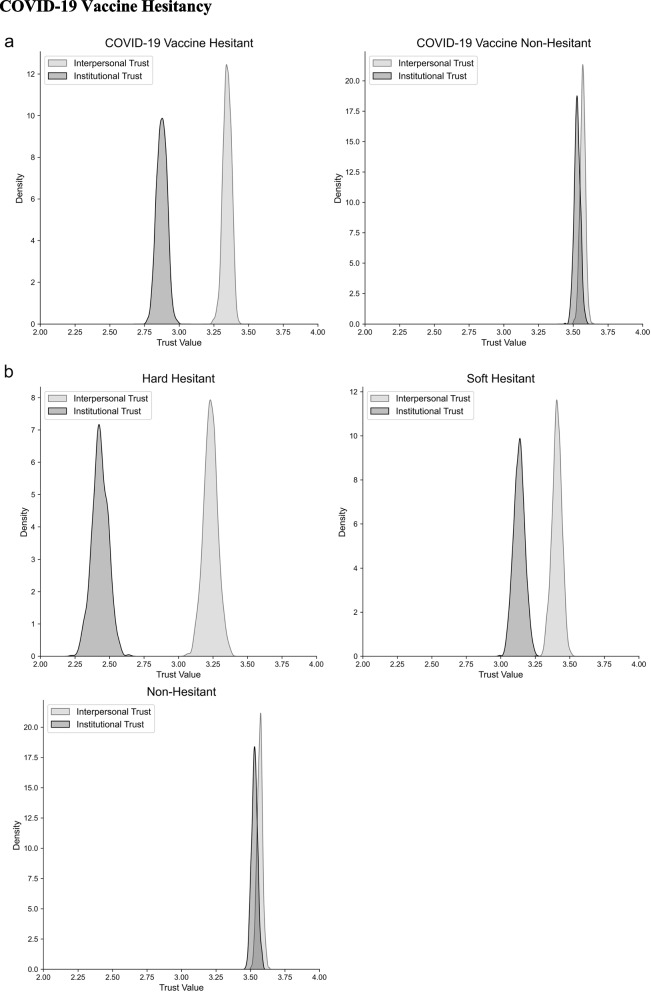
**a** Individuals exhibiting COVID-19 vaccine hesitancy showed significant differences between levels of interpersonal trust and institutional trust, whereas those with no COVID-19 vaccine hesitancy did not. **b** The differences in interpersonal versus institutional trust increase as hesitancy increases, with hard hesitant individuals exhibiting significantly larger delta than soft-hesitant individuals, who in turn exhibit significantly higher delta than non-hesitant individuals

We segregated trust scores within each group into interpersonal and institutional trust components and computed the difference between the two (Fig. 1a). The hesitant group exhibited a significant discrepancy between interpersonal (M = 3.343, SD = 0.029) and institutional trust (M = 2.879; t(567) = 9.951, *p* < 0.001), while this distinction was not observed in the non-concerned group. Further comparison of the two differences revealed that the concerned group exhibited a significantly larger disparity between institutional and interpersonal trust (M = -0.467, SD = 0.820) than the non-concerned group (M = 0.038, SD = 0.648; t(1539) = *p* < 0.001).

We conducted an eta correlation ratio analysis to explore the relationship between COVID-19 vaccine concerns as a categorical variable and each type of trust as a continuous variable. The results indicated a stronger eta correlation ratio between institutional trust and hesitancy, compared to that between interpersonal trust and hesitancy (η = 0.256 for interpersonal vs. η = 0.509 for institutional trust). The bootstrapped 95% confidence interval for the difference in correlations ranged from -0.304 to -0.197, suggesting that institutional deficits in institutional trust had a significantly stronger association with hesitancy.

Finally, we split participants into three groups: hard hesitants (score = 4 on the measure as defined above); soft hesitants (score = 2 or score = 3) and non-hesitants (score = 1) in order to determine the extent to which institutional trust deficits might relate to different hesitancy levels (Fig. 1b). Non-hesitant individuals showed no significant difference between interpersonal and institutional trust (M = 3.567, SD = 0.019 for interpersonal; M = 3.530, SD = 0.023 for institutional; t(970) = 1.294, p = 0.196). Soft hesitant individuals and hard hesitant individuals did show significant differences between types of trust (t(359) = 5.099, *p* < 0.001 for soft-hesitant and t(206) = 1.190, *p* < 0.001 for hard hesitant individuals). An analysis of the delta between trust types in soft-hesitant and hard-hesitant individuals revealed a significant difference between the two. Our results suggest that as hesitancy grows, mistrust in institutions has a significantly stronger association with hesitancy.

Next, we sought to investigate the association between high and low levels of COVID-19 vaccine concerns and varying degrees of interpersonal and institutional trust. As a reminder, this construct (Additional file 1: Appendix B, Instrument B4) refers to a series of potential concerns about the COVID-19 vaccine and its production. Participants were categorized into concerned and non-concerned groups based on whether they had a score of either more than or less than/equal to 0.17 (the median) in that measure. This analysis revealed that concerned individuals displayed overall reduced trust (M = 3.14, SD = 0.69) compared to their non-concerned counterparts (M = 3.55, SD = 0.59; t(1539) = -12.54, *p* < 0.001).

**Fig. 2:**
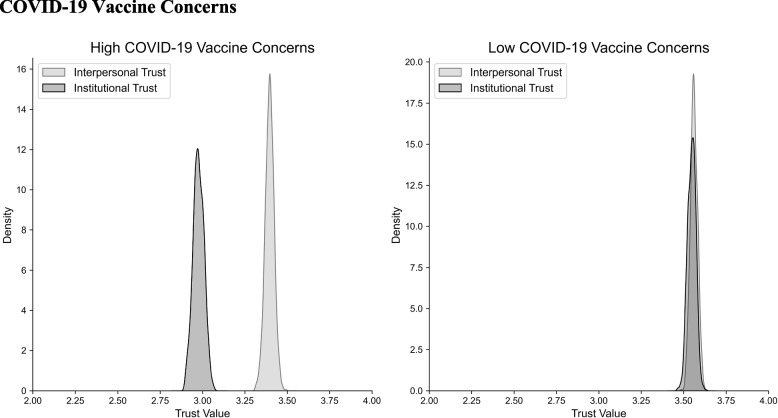
Individuals exhibiting COVID-19 vaccine concerns showed significant differences between levels of interpersonal and institutional trust, whereas those with no COVID-19 vaccine concerns did not

Subsequently, we segregated trust scores within each group into interpersonal and institutional trust components and computed the difference between the two (Fig. 2). The concerned group exhibited a significant discrepancy between interpersonal (M = 3.393, SD = 0.025) and institutional trust (M = 2.978, SD = 0.033; t(700) = 1.124, *p* < 0.001), while this distinction was not observed in the non-concerned group. Further comparison of the two differences revealed that the concerned group exhibited a significantly larger disparity between institutional and interpersonal trust (M = -0.417, SD = 0.791) than the non-concerned group (M = 0.02, SD = 0.651; t(1539) = 1.822, *p* < 0.001).

Lastly, we conducted an eta correlation ratio analysis to explore the relationship between COVID-19 vaccine concerns as a categorical variable and each type of trust. The results indicated a stronger correlation ratio with institutional trust relative to interpersonal trust (η = 0.227 for interpersonal vs. η = 0.496 for institutional trust). The bootstrapped 95% confidence interval for the difference in correlations ranged from -0.321 to -0.217, indicating that the difference was statistically significant.

We explored the association between high and low levels of general vaccine hesitancy and varying degrees of interpersonal and institutional trust. Participants were categorized into hesitant and non-hesitant groups based on whether they had a score of 0 or more than 0 (the median) in that measure. This analysis revealed that general vaccine hesitant individuals displayed overall reduced trust (M = 2.85, SD = 0.71) compared to their low concern counterparts (M = 3.41, SD = 0.65; t(1539) = 903, *p* < 0.001).

**Fig. 3:**
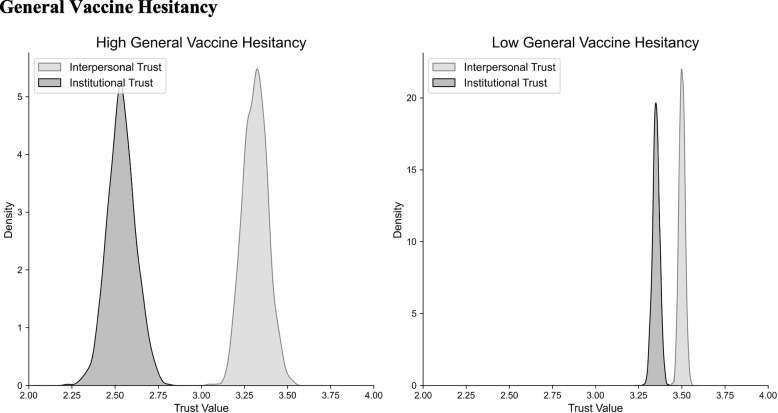
Individuals exhibiting general vaccine hesitancy showed significant differences between levels of interpersonal and institutional trust. Those with no general vaccine hesitancy also showed significantly lower institutional versus interpersonal trust. However, the delta in the high hesitancy group was significantly higher than the delta in the low hesitancy group

We again segregated trust scores within each group into interpersonal and institutional trust components and computed the difference between the two (Fig. 3). The hesitant group exhibited a significant discrepancy between interpersonal (M = 3.319, SD = 0.070) and institutional trust (M = 2.537, SD = 0.079; t(117) = 7.404, *p* < 0.001), as did the non-hesitant group (M = 3.499, SD = 0.017 for interpersonal; M = 3.351, SD = 0.021 for institutional; t(1420) = 5.465), *p* < 0.001). However, a comparison of the two differences revealed that the hesitant group exhibited a significantly larger disparity between institutional and interpersonal trust (M = -0.781, SD = 0.869) than the non-hesitant group (M = -0.148, SD = 0.713; t(1539) = 7.731, *p* < 0.001).

Lastly, we conducted an eta correlation ratio analysis to explore the relationship between general vaccine hesitancy as a categorical variable and each type of trust. The results indicated a significantly stronger correlation ratio with institutional trust relative to interpersonal trust (η = -0.229 for interpersonal vs. η = -0.394 for institutional trust). The bootstrapped 95% confidence interval for the difference in correlations ranged from -0.264 to -0.067, indicating that the difference was statistically significant.

Next, we sought to investigate the association between high and low levels of COVID-19 conspiracy thinking and varying degrees of interpersonal and institutional trust. Participants were categorized into high and low conspiracy thinking groups based on the median (0). Our analysis revealed that high conspiracy thinking individuals displayed overall reduced trust (M = 3.13, SD = 0.69) compared to their low conspiracy thinking counterparts (M = 3.58, SD = 0.56; t(1539) = -14.24, *p* < 0.001).

**Fig. 4:**
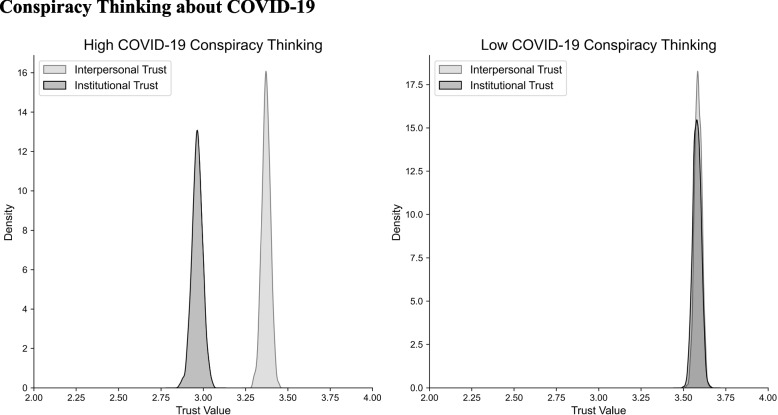
Individuals exhibiting high COVID-19 conspiratorial beliefs showed significant differences between levels of interpersonal and institutional trust, whereas those without COVID-19 vaccine hesitancy did not

Subsequently, we segregated trust scores within each group into interpersonal and institutional trust components, and computed the difference between the two (Fig. 4). The high conspiracy group exhibited a significant discrepancy between interpersonal (M = 3.372, SD = 0.025) and institutional trust (M = 2.963, SD = 0.032; t(728) = 9.995, *p* < 0.001), while this distinction was not observed in the low conspiracy group. Further comparison of the two differences revealed that the high conspiracy group exhibited a significantly larger disparity between institutional and interpersonal trust (M = -0.407, SD = 0.794) than the low-conspiracy group (M = 0.007, SD = 0.643; t(1539) = 1.809, *p* < 0.001).

Lastly, we conducted an eta correlation ratio analysis to explore the relationship between COVID-19 vaccine concerns as a categorical variable and each type of trust. The results indicated a stronger correlation ratio with institutional trust (η = -0.516) compared to interpersonal trust (η = -0.258). The bootstrapped 95% confidence interval for the difference in correlations ranged from -0.305 to -0.208, indicating that the difference was statistically significant.

Finally, we examined whether institutional trust would be associated with COVID-19 vaccination status. We saw that the overall level of trust is lower in the unvaccinated versus the vaccinated (M = 2. 98, SD = 0.72 versus M = 3.51, SD = 0.59; t(1539) = 14.76; *p* < 0.001).

**Fig. 5:**
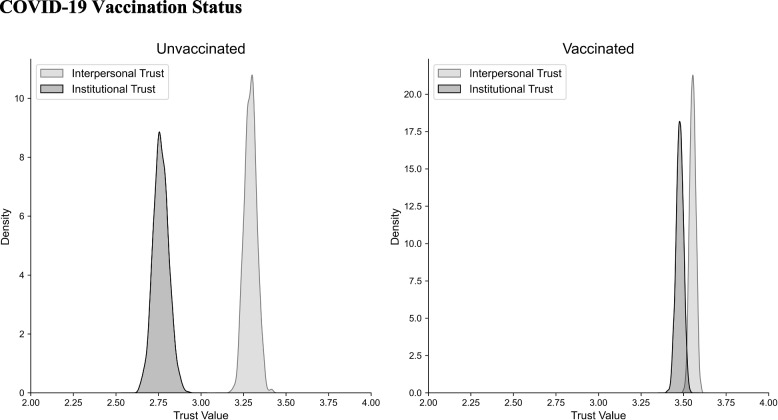
Individuals that were unvaccinated for COVID-19 showed significant differences between levels of interpersonal and institutional trust. Vaccinated individuals also showed significantly lower institutional versus interpersonal trust. However, the delta in the high hesitancy group was significantly higher than the delta in the low hesitancy group

When segregating the trust scores within each group into interpersonal and institutional trust components (Fig. 5), we found the unvaccinated group exhibited a significant discrepancy between interpersonal (M = 3.289, SD = 0.037) and institutional trust (M = 2.767, SD = 0.042; t(408) = 9.176, *p* < 0.001), as did the vaccinated group (M = 3.556, SD = 0.017 for interpersonal; M = 3.478, SD = 0.022 for institutional; t(1129) = 2.801, *p* < 0.01). However, while both groups showed significant difference in interpersonal versus institutional trust, a comparison of the two differences revealed the unvaccinated group exhibited a significantly larger disparity between institutional and interpersonal trust (M = -0.524, SD 0.840) than the vaccinated group (M = -0.078, SD = 0.670; t(1539) = 9.697, *p* < 0.001).

Lastly, we conducted an eta correlation ratio analysis to explore the relationship between vaccination status and each type of trust. The results indicated a significantly stronger association of vaccination status with institutional trust relative to interpersonal trust (η = 0.248 for interpersonal vs. η = 0.443 for institutional trust). The bootstrapped 95% confidence interval for the difference in correlations ranged from -0.246 to -0.145, indicating that the difference was statistically significant.

### Summary graphs

**Fig. 6:**
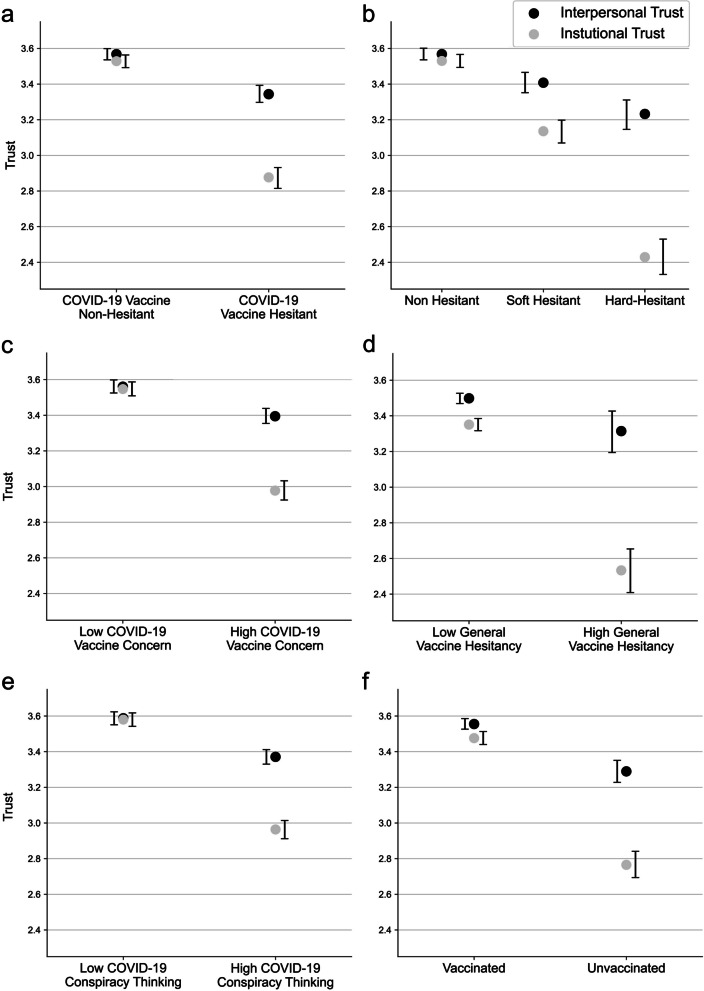
Summary plot of the comparisons between interpersonal and institutional trust across **a** low versus high COVID-19 vaccine hesitancy; **b** non-hesitant, soft hesitant and hard hesitant in regards to COVID-19 vaccines; **c** low and high COVID-19 vaccine concerns; **d** low and high general vaccine hesitancy; **e** low and high COVID-19 conspiracy thinking; **f** vaccinated and unvaccinated. Error bars represent the 5th and 95th percentile of the bootstrapped means as described above

Given the hypothesized relatedness of the constructs we used (COVID-19 vaccine hesitancy, COVID-19 vaccine concerns, general vaccine hesitancy, COVID-19 conspiracy thinking and COVID-19 vaccination status), a summary graph of comparisons was created (Fig. 6). As can be seen, plotting the relationships between each of these constructs and each type of trust shows that the two trust scores tend to be far more aligned in non-hesitant individuals across all measures relative to their counterparts, where institutional distrust is a clear driver of the overall lower trust levels.

## Discussion

We set out to test whether individuals exhibiting various vaccine hesitancy related beliefs and behaviors have a different trust profile from those who do not. Consistent with our hypothesis, we found that vaccine hesitancy and other related factors (COVID-19 vaccine concerns, COVID-19 conspiracy thinking, general vaccine hesitancy, COVID-19 vaccine hesitancy and COVID-19 vaccination status) are associated with lower levels of trust specific to institutions.

Our analysis revealed a distinct relationship between trust in institutions, such as scientists and the WHO, and vaccine hesitancy. This relationship was first measured using an eta correlation ratio between 15 different entities and our five vaccine hesitancy related measures. Results revealed that all of the strongest relationships were between the five measures and distrust in institutions; in particular, distrust in scientists and vaccination status (η = 0.564), followed by distrust in scientists and COVID-19 vaccine hesitancy (η = 0.498), and distrust in the WHO and COVID-19 vaccination status (η = 0.457).

Next, we grouped trust in interpersonal entities (Cronbach's Alpha of 0.802) and trust in institutions (Cronbach's Alpha of 0.894) and found a significant discrepancy in levels of interpersonal and institutional trust among individuals exhibiting COVID-19 vaccine hesitancy. While hesitant individuals displayed overall reduced trust (M = 3.06, SD = 0.70) compared to non-hesitant counterparts (M = 3.54, SD = 0.58; t(1539) = -14.54, *p* < 0.001), we observed that the hesitant group exhibited a significant discrepancy between interpersonal (M = 3.343, SD = 0.029) and institutional trust (M = 2.879; t(567) = 9.951, *p* < 0.001). This discrepancy was not observed in the non-hesitant group, suggesting that institutional trust deficits may be more closely associated with vaccine hesitancy. Importantly, we found that hesitant individuals also had lower levels of interpersonal trust compared to their non-hesitant counterparts. We performed the same analyses across all of our measures and found the difference in institutional versus interpersonal trust was significantly larger for groups exhibiting higher COVID-19 vaccine concerns, higher COVID-19 conspiracy thinking, higher general vaccine hesitancy and lack of COVID-19 vaccination (Fig. 6).

Our findings are generally aligned with the existing literature on the relationship between trust and vaccine hesitancy. For example, in a study of over 13,000 people and 19 countries, Lazarus and colleagues [59] found that higher levels of trust in information from government sources was linked to a higher likelihood to accept a vaccine. Interestingly, the same study found that trusting information from the government was also related to a higher propensity to positively respond to vaccine information coming from their employer. This relationship between trust in one institution and another is not surprising in the context of our findings, which include both governmental and non-governmental institutions, but raises the possibility that changes in trust toward one institution may influence trust toward other seemingly unrelated institutions.

This is reflected in past research, which has reported trust in specific institutions as important for vaccine uptake, but has done so in a way that does not unify these findings under the broader umbrella of institutional trust. For example, research by Palamenghi and colleagues [60] on 968 Italian citizens early on in the pandemic revealed similar insights, concluding that trust in scientific institutions is a key factor in vaccine uptake. In a non-COVID-19 vaccination context, Schmid and colleagues [61] found that influenza vaccine hesitancy is strongly related to distrust in health authorities. Similarly, research by Schwarzinger and colleagues [62] reported that trust in health policy and services was a key factor in vaccine hesitancy, while Dror and colleagues [63] suggested that distrust in preventative healthcare is a key factor. While our research supports findings like these, the relatively high Cronbach’s alpha (0.894) we reported within institutional trust measures suggests that a broader view of this construct may be warranted. On this view, past research proposing trust in large entities as significantly related to vaccine uptake should perhaps be interpreted, to some extent, as reporting on different dimensions of the same construct—institutional trust. Such a view is broadly aligned with past thinking on trust in institutions, which suggests that it is defined by the power-dynamic between individuals and institutions. However, future research is required to understand the extent to which “institutional trust” can be thought of as more than just a sum of its parts, as well as the effect this may have on vaccine hesitancy.

Our findings revealed that while lower general levels of trust were present in vaccine hesitant individuals across all measures, there was a significantly larger difference between interpersonal and institutional trust in that group than in the non-hesitant group across all five measures. Furthermore, in the case of COVID-19 conspiracy thinking and COVID-19 vaccine hesitancy, results showed that there is a significant difference between institutional and interpersonal trust for the hesitant but not for the non-hesitant group (M = -0.467 versus M = 0.038, respectively, for COVID-19 vaccine hesitancy; M = -0.407 versus M = -0.007, respectively, for COVID-19 conspiracy thinking). Again, these findings suggest that there is an institutional trust deficit specific to those individuals exhibiting vaccine hesitancy related-attitudes.

Our results indicate that overall levels of trust (across interpersonal and institutional entities) are lower in vaccine hesitant individuals. Consistent with past research [64], we found lower levels of interpersonal trust in individuals who scored higher on measures of vaccine hesitancy. However, consistent with past work by Goldenberg [16], we found institutional trust to be significantly more strongly associated with vaccine hesitancy than interpersonal trust. This lack of balance between institutional and interpersonal trust, which we did not observe in the non-hesitant group, raises the possibility that hesitant individuals may be basing their vaccine decisions on alternative information sources. For example, a lack of trust in institutions may push a vaccine hesitant individual to instead rely on advice sourced from their social group. Past research has shown that strong ties tend to exist between vaccine hesitant individuals [65], further supporting the idea that distrust in institutions is likely to create vaccine hesitant echo-chambers. Given that vaccine-hesitant individuals in our sample have a higher relative trust toward non-institutional entities, our findings add to an increasingly supported narrative suggesting that vaccine hesitancy is predominantly a social phenomenon related to trust rather than a cognitive phenomenon related to deficits in decision making.

### Public health implications

The results presented here suggest that current public health strategies that are used to increase vaccine uptake might be effective for individuals who have higher levels of institutional trust, but have a weaker and possibly counterproductive effect on groups with low institutional trust. Given that marginalized communities are the most likely to have lower levels of institutional trust [16], they likely present the biggest challenge for public health professionals.

Past research has shown that the way information about vaccines is presented is critical to ensure a positive effect on vaccine uptake [66–68]. Our research builds on this by suggesting that while fast dissemination of critical information by large entities such as the WHO is important (in particular during a pandemic that is extremely dynamic), lower trust levels on the part of vaccine hesitant individuals toward institutions indicate that, beyond a certain vaccine uptake point, it may be beneficial to emphasize a more community-focused strategy that leverages strong ties. The extent to which different communication strategies (broad versus community focused) are compatible is an important question for future research.

Importantly, in the context of a time-bound vaccination campaign (such as the ones we are likely to encounter in future pandemics), as time goes on, the target population for public health messages shifts from one that is predominantly vaccine acceptant to one that is predominantly vaccine hesitant. Because the attitudes toward institutions between these groups are drastically different, it is very likely that in order to optimize vaccine uptake, messaging strategies must change as the target audience does. While at the beginning we can assume that only a minority of the target are vaccine hesitant and an institution-derived diffusion strategy (e.g., the WHO) is likely to be effective, as vaccination rates climb, a higher and higher percentage of the target group is composed of vaccine hesitant individuals with low institutional trust for whom a message from the WHO is likely to be counterproductive. Therefore, an inflection point likely exists, after which the dominant strategy should be changed. It is worth noting here that while differences in institutional trust were significantly higher in hesitant compared to non-hesitant individuals, hesitant individuals did also show lower interpersonal trust scores. Therefore, while public health strategies targeting hesitant individuals may be generally more successful by shifting toward strong ties at an inflection point, even those strong ties may be less effective in increasing vaccine uptake relative to the strong ties of non-hesitant individuals.

While it is difficult to say where this inflection point lies or what precise strategies should be used before and after it occurs, public health messaging architects should be aware that the very same message is likely to be received quite differently as the composition of their audience changes to include predominantly vaccine hesitant individuals. Based on these findings, it seems that public health messaging should be based on a closer monitoring of the target audience’s “trust balance”—i.e. the extent to which their total trust is based on interpersonal versus institutional relationships.

Our findings, along with past research on institutional trust, suggest that a messaging strategy originating in strong ties may be more effective with hesitant individuals. Therefore, we encourage policymakers to pre-empt or reduce messaging that evokes out-group feeling, distinctiveness, or ‘Othering’ of those who remain unvaccinated. Effective strategies may include outreach through local community networks and familiar contacts. A “chorus” of peer-led voices is more likely to be welcomed over top-down approaches.

In addition to shifting messaging strategies from ones focused on institutions to ones that leverage strong ties, in the context of the importance of institutional trust, public health agencies have a responsibility to attempt to rebuild trust in the individuals that have lost it. As noted by Goldenberg [16, 28], the understanding of institutional trust as the crux of the vaccine hesitancy problem creates a strong need for conciliation between public health agendas and the needs of communities. Therefore, while immediate strategies should focus on strong ties, longer term efforts should focus on engaging communities in an equitable and transparent manner that is sensitive to the historical causes of mistrust and aimed at correcting systemic inequities.

### Limitations

While the data presented in this paper raise the possibility that people with different vaccine attitudes and behaviors show differential sensitivity to institutions compared to individuals when building trust, our findings come with a number of caveats. First and foremost, while our study revealed a strong link between trust profiles and vaccine hesitancy-related measures, the cross-sectional design prevents us from drawing any causal conclusions about the relationships between these variables.

#### Timing

The COVID-19 pandemic was profoundly disruptive. Given that the data collected in this study was from that time period, it is possible that factors relating to the pandemic affect trust-size sensitivity. For example, people who are generally low on the vaccine hesitancy continuum may show attitudes and behaviors that exaggerate their baseline level of doubt because of the higher level of uncertainty associated with the pandemic—for example, due to factors such as the rapid development of the pandemic, the fast-changing evidence base, changes in vaccine policies and a perceived rush in developing the vaccines.

#### Geography

Data for this study was collected in Canada, and vaccine attitudes vary greatly among some countries. Therefore, any application of the insights reported here should make efforts to contextualize and validate these insights in a localized sample. This is particularly important given the following factors. First, Canada’s publicly-funded universal healthcare system is likely to raise a distinct set of problems of access and affordability. Second, the relationship between citizens and the healthcare system is different compared to countries such as the United States, and this is very likely to affect how free-of-charge vaccines such as the ones against COVID-19 are perceived. In other words, free vaccines may be perceived differently in a place where healthcare is generally free compared to one where it is not.Third, Canada’s multicultural society provides an interesting backdrop against which to assess institutional trust. As shown by past research, deficits in institutional trust are more common in particular communities. Therefore, our findings should be confirmed with closer studies of those communities, in a transparent and collaborative fashion, before they are directly applied to create messaging strategies that target vaccine uptake.

#### Reported trust

While our instrument collects reported trust data about a number of different groups, we do not actually measure trust in any non-self-reported manner. While past research on trust has taken a similar approach, we recognize that self-report may not always translate into behavior. Therefore, those looking to apply this research to predict behaviors relying on trust should validate that the effects apply to those behaviors as well.

### Future directions

While our work shows the relationship between trust and various vaccine-related attitudes and behaviors, it does not characterize the entities to be trusted beyond “interpersonal” and “institutional”. Despite relatively high Cronbach’s Alphas for each of these groups, it is unlikely that this is truly a binary distinction. Future work looking to expand on these insights should test whether more nuanced aspects of an institution (for example, feeling of connectedness to that institution, perceived transparency, perceived interconnectedness, etc.) might be better predictors of trust sensitivity than institution status alone.

Furthermore, while we looked at the relationship between trust and measures of vaccine hesitancy, it is likely that a number of mediating variables exist between these two measures. Future work looking to understand how levels of institutional trust might relate to vaccine-related attitudes should look at possible psychological parameters that may mediate trust sensitivity, as understanding those could make this work generalizable to a large number of contexts.

Finally, in the context of past work on institutional trust and vaccine hesitancy [16], future work should endeavor to better understand how the findings gleaned from our study relate to individual communities. Deficits in institutional trust (and the vaccine hesitancy outcomes that follow) are more strongly felt in marginalized communities that have historical reasons for that mistrust. Therefore, efforts to understand how trust might be repaired is critical. Barring that, investigating how public health strategies can best circumvent institutions when targeting vaccine uptake in those communities, is also important.

## Conclusion

Interpersonal and institutional trust are distinct concepts [69]. Importantly, as we shift from one to the other, typical social cues (and the related body of scientific research) become less relevant and a different set of conditions must be met for trust to form [70].

We set out to find out whether institutional trust is distinctly related to attitudes and behaviors that the vaccine hesitant tend to exhibit—COVID-19 vaccine hesitancy, COVID-19 vaccine concerns, general vaccine hesitancy, COVID-19 conspiracy thinking and COVID-19 vaccination. The data presented in this paper shows that all of these outcome variables are more strongly related to institutional rather than interpersonal trust levels.

### Supplementary Information

**Additional file 1.**

## Data Availability

The datasets used during the current study are available from the corresponding author on reasonable request.
