# Supplementary material for: Institutional trust is a distinct construct related to vaccine hesitancy and refusal
Source: BMC Public Health. 2023 Dec 12;23:2481. doi: 10.1186/s12889-023-17345-5 (PMC10714562; doi:10.1186/s12889-023-17345-5)
Supplement: Supplementary file 1 — Additional file 1. [file 12889_2023_17345_MOESM1_ESM.docx]

## **Appendix A: Sample Characteristics**

The table below presents sample characteristics across the 1541 Canadians over the age of 18 who completed our survey. Of those, 1165 (76%) had received at least two doses of an approved COVID vaccine, 376 (25%) were unvaccinated vaccine hesitant. The mean age of our sample was 46.4 years (range: 18 - 87). The majority of our sample were women (57%), white (71%), and had at least a high school education (85%). A small portion of our sample self-identified as Indigenous (6%).

Table 2: Sociodemographic characteristics of the sample

**______________________________________________________________________**

|  | **Total** | | **Vaccinated** | |
| --- | --- | --- | --- | --- |
|  | **N** | **%** | **N** | **%** |
|  | 1541 | 100% | 1165 | 76% |
| **Age** |  | | | |
| 18-25 | 159 | 10% | 110 | 69% |
| 26-35 | 304 | 20% | 199 | 65% |
| 36-45 | 236 | 15% | 158 | 67% |
| 46-55 | 281 | 18% | 219 | 78% |
| 56+ | 561 | 36% | 479 | 85% |
| **Gender** |  | | | |
| Man | 653 | 42% | 515 | 79% |
| Woman | 879 | 57% | 642 | 73% |
| NA/Other | 9 | 1% | 8 | 89% |
| **Ethnicity** |  | | | |
| Black | 53 | 3% | 31 | 58% |
| East Asian | 91 | 6% | 70 | 77% |
| Indigenous | 89 | 6% | 60 | 67% |
| Latino | 25 | 2% | 19 | 76% |
| Middle Eastern | 27 | 2% | 21 | 78% |
| South Asian | 70 | 5% | 62 | 89% |
| Southeast Asian | 20 | 1% | 19 | 95% |
| White | 1091 | 71% | 829 | 76% |
| Do not know | 16 | 1% | 11 | 69% |
| Prefer not to answer | 25 | 2% | 17 | 68% |
| Other | 66 | 4% | 47 | 71% |
| **Indigenous Status** |  | | | |
| Yes, First Nations | 42 | 3% | 28 | 67% |
| Yes, Métis | 42 | 3% | 28 | 67% |
| Yes, Inuk (Inuit) | 5 | 0% | 4 | 80% |
| No, not an Indigenous person | 1419 | 92% | 1084 | 76% |
| I prefer not to answer | 32 | 2% | 20 | 63% |
| **Province** |  | | | |
| Alberta | 152 | 10% | 109 | 72% |
| British Columbia | 207 | 13% | 161 | 78% |
| Manitoba | 46 | 3% | 33 | 72% |
| New Brunswick | 35 | 2% | 20 | 57% |
| Newfoundland and Labrador | 20 | 1% | 15 | 75% |
| Northwest Territories | 0 | 0% | 0 | NA |
| Nova Scotia | 40 | 3% | 31 | 78% |
| Nunavut | 0 | 0% | 0 | NA |
| Ontario | 624 | 40% | 475 | 76% |
| Prince Edward Island | 7 | 0% | 5 | 71% |
| Québec | 368 | 24% | 284 | 77% |
| Saskatchewan | 42 | 3% | 32 | 76% |
| Yukon | 0 | 0% | 0 | NA |
| Other | 0 | 0% | 0 | NA |
| **Education** |  | | | |
| Less than high school | 53 | 3% | 33 | 62% |
| Completed some high school | 177 | 11% | 113 | 64% |
| High school graduate or equivalent | 445 | 29% | 332 | 75% |
| Technical college, community college or CEGEP | 386 | 25% | 297 | 77% |
| Completed some university, but no degree | 112 | 7% | 90 | 80% |
| University graduate | 226 | 15% | 184 | 81% |
| Completed some post-graduate school, but no degree | 44 | 3% | 30 | 68% |
| Completed post-graduate school | 98 | 6% | 86 | 88% |
| **Income (CAD)** |  | | | |
| Less than $10,000 | 129 | 8% | 82 | 64% |
| $10,000 to $19,999 | 315 | 20% | 231 | 73% |
| $20,000 to $29,999 | 365 | 24% | 268 | 73% |
| $30,000 to $39,999 | 213 | 14% | 162 | 76% |
| $40,000 to $49,999 | 109 | 7% | 81 | 74% |
| $50,000 to $59,999 | 71 | 5% | 55 | 77% |
| $60,000 to $69,999 | 60 | 4% | 46 | 77% |
| $70,000 to $79,999 | 52 | 3% | 44 | 85% |
| $80,000 to $89,999 | 30 | 2% | 23 | 77% |
| $90,000 to $99,999 | 48 | 3% | 40 | 83% |
| $100,000 to $199,999 | 97 | 6% | 88 | 91% |
| $200,000 or more | 11 | 1% | 10 | 91% |
| Prefer not to answer | 41 | 3% | 35 | 85% |

**______________________________________________________________________**

##

## **Appendix B: Instruments Used**

**B1: COVID-19 Vaccine Hesitancy**

COVID-19 vaccine hesitancy was determined by assessing the answers to the following question:

*B1.1: Knowing that vaccinations against COVID-19 have begun, have you received the COVID-19 vaccination?*

*1: Yes*

*2: No*

*B1.2: When the COVID-19 vaccination becomes available to you, would you get vaccinated or not?*

*1: Yes, I would get a vaccination as soon as one became available to me*

*2: Yes, I would eventually get a vaccination, but would wait a while first*

*3: No, I would not get a COVID-19 vaccination*

*4: Not sure*

*B1.3: Thinking back, how hesitant were you about a COVID-19 vaccination prior to receiving one?*

*1: Was not hesitant*

*2*

*3: Was neither hesitant nor not hesitant*

*4*

*5: Was extremely hesitant*

**B2: Trust**

Trust in various groups of varying sizes was determined by assessing the answers to the following question:

*B2.1: On a scale of one to five, in general how much do you trust the following people and institutions where one being not at all and five being completely (1: Not at all; 2: Little; 3: Neither trust nor don’t; 4: Somewhat; 5: Completely):*

*Family*

*Friends*

*Acquaintances*

*Classmates*

*Co-workers*

*Roommates*

*The federal government*

*The local government*

*The World Health Organization (WHO)*

*The healthcare system*

*The police*

*Scientists/COVID-19 Researchers*

*Physicians/Medical doctors*

*Mainstream Media (i.e., news outlets)*

*Pharmaceutical companies*

**B3: Conspiratorial Thinking**

Conspiratorial thinking in relation to COVID-19 was determined by calculating an equally weighted average to answers of the following questions:

*B3.1: How much do you agree with the following statements (1: Strongly disagree; 2: Somewhat disagree; 3: Neither agree nor disagree; 4: Somewhat agree; 5: Strongly agree):*

*1. Governing bodies knew about COVID-19 long before the public*

*2. There is a cure for COVID-19, and it is being withheld from the public*

*3. COVID-19 was purposefully created by a larger governing body*

*4. 5G mobile networks are related to the spread of COVID-19*

*5. COVID-19 was created/engineered in a lab*

*6. COVID-19 is a hoax and scientists are lying to us*

*7. A large governing body is planning to implant microchips for global surveillance through the COVID-19 vaccination plan*

*8. George Soros has played a role in creating the pandemic or is benefiting from it in some way*

*9. Governments are exaggerating the seriousness of the COVID-19 situation in order to control the population*

*1. Hospitals are registering every death from other causes as a COVID death if the person had tested positive in the las 28 days*

**B4: COVID-19 Vaccine Concerns**

COVID-19 vaccine concern were determined by calculating an equally weighted average to answers of the following questions:

B4.1: *What concerns, if any, do you have about the COVID-19 vaccine? (checkboxes, values 0-1):*

*1. I am concerned about side effects from a coronavirus vaccine*

*2. I am concerned the coronavirus vaccine will not be effective*

*3. This vaccine’s production has been rushed and is therefore not safe*

*4. The vaccine will somehow be used to infringe on my privacy*

*5. I believe the vaccine will be used to harm the population*

*6. I have no concerns about the COVID-09 vaccine*

**B5: General Vaccine Hesitancy**

General vaccine hesitancy was determined by calculating an equally weighted average to answers of the following questions:

B5.1: *What concerns, if any, do you have about the COVID-19 vaccine? (checkboxes, values 0-1):*

*1. In general, vaccines are not safe*

*2. In general, vaccines are not necessary*

##

**Appendix D: Trust delta comparisons**

Table 3: Comparison of the trust deltas (institutional - interpersonal trust) between the high and low hesitant groups.

**______________________________________________________________________**

| **Group 1** | **Group 2** | **M1** | **SD1** | **M2** | **SD2** | **T Statistic** | **p-value** |
| --- | --- | --- | --- | --- | --- | --- | --- |
| COVID-19 Vaccine Hesitant | COVID-19 Vaccine Non-Hesitant | -.467 | .819 | -.038 | .647 | -11.343 | <.001 |
| High COVID-19 Vaccine Concerns | Low COVID-19 Vaccine Concerns | -.417 | .790 | -.013 | .650 | -11.008 | <.001 |
| High General Vaccine Hesitancy | Low General Vaccine Hesitancy | -.781 | .865 | -.148 | .713 | -9.131 | <.001 |
| High COVID-19 Conspiracy Thinking | Low COVID-19 Conspiracy Thinking | -.407 | .793 | -.007 | .642 | -1.927 | <.001 |
| Unvaccinated | Vaccinated | -.524 | .839 | -.078 | .670 | -1.763 | <.001 |

**______________________________________________________________________**
